# Use of Estimating Equations for Dosing Antimicrobials in Patients with Acute Kidney Injury Not Receiving Renal Replacement Therapy

**DOI:** 10.3390/jcm7080211

**Published:** 2018-08-11

**Authors:** Linda Awdishu, Ana Isabel Connor, Josée Bouchard, Etienne Macedo, Glenn M. Chertow, Ravindra L. Mehta

**Affiliations:** 1 Division of Clinical Pharmacy, UCSD Skaggs School of Pharmacy and Pharmaceutical Sciences, San Diego, CA 92093, USA; lehans158@msn.com; 2 Department of Medicine, Division of Nephrology, UCSD School of Medicine, San Diego, CA 92093, USA; emacedo@ucsd.edu (E.M.); rmehta@ucsd.edu (R.L.M.); 3 Department of Medicine, University of Montreal, Montreal, QC H3T 1J4, Canada; joseebouchard123@yahoo.ca; 4 Department of Medicine, Division of Nephrology, Stanford University School of Medicine, Palo Alto, CA 94034, USA; gchertow@stanford.edu

**Keywords:** acute kidney injury, Cockcroft Gault, Jelliffe, MDRD, drug dosing, antimicrobials

## Abstract

Acute kidney injury (AKI) can potentially lead to the accumulation of antimicrobial drugs with significant renal clearance. Drug dosing adjustments are commonly made using the Cockcroft-Gault estimate of creatinine clearance (CLcr). The Modified Jelliffe equation is significantly better at estimating kidney function than the Cockcroft-Gault equation in the setting of AKI. The objective of this study is to assess the degree of antimicrobial dosing discordance using different glomerular filtration rate (GFR) estimating equations. This is a retrospective evaluation of antimicrobial dosing using different estimating equations for kidney function in AKI and comparison to Cockcroft-Gault estimation as a reference. Considering the Cockcroft-Gault estimate as the criterion standard, antimicrobials were appropriately adjusted at most 80.7% of the time. On average, kidney function changed by 30 mL/min over the course of an AKI episode. The median clearance at the peak serum creatinine was 27.4 (9.3–66.3) mL/min for Cockcroft Gault, 19.8 (9.8–47.0) mL/min/1.73 m^2^ for MDRD and 20.5 (4.9–49.6) mL/min for the Modified Jelliffe equations. The discordance rate for antimicrobial dosing ranged from a minimum of 8.6% to a maximum of 16.4%. In the event of discordance, the dose administered was supra-therapeutic 100% of the time using the Modified Jelliffe equation. Use of estimating equations other than the Cockcroft Gault equation may significantly alter dosing of antimicrobials in AKI.

## 1. Introduction

Acute kidney injury (AKI) has been reported to occur in approximately 6% of hospitalized patients [1]. Among patients admitted with AKI, infection is present in approximately 18% [2]. AKI is particularly common among critically ill patients and has been associated with increased morbidity and significant in-hospital mortality [2,3]. A decline in kidney function can potentially lead to the accumulation of antimicrobial and other therapeutic agents, with resultant adverse effects [4]. An accurate assessment of kidney function is important in order to optimize drug administration in this population [5,6,7]. 

The most accurate way to determine glomerular filtration rate (GFR) in chronic kidney disease (CKD) is by formal measurement using an intravenous injection of inulin or a radioisotope and subsequently collecting urine and serum samples at timed intervals [8,9]. However, the direct measurement of GFR is cumbersome, expensive and time consuming, and rarely performed in the acute hospital setting. These procedures are even more complicated in AKI. Pharmacists generally employ the Cockcroft-Gault (CG) equation to estimate kidney function, altering either or both the dose and frequency of drugs based on varying degrees of evidence in the setting of impaired kidney function and/or dialysis [10,11,12,13,14,15,16]. 

Several newer GFR estimating equations have been developed and used widely in epidemiological studies and clinical practice, including the Modification of Diet in Renal Disease (MDRD) study equation and the CKD-EPI equation. These equations were derived from varying populations who generally had stable kidney function. For example, the CG equation was derived from a hospitalized population including predominantly Caucasian men with stable serum creatinine concentrations (Scr) [17]. The MDRD study and CKD-EPI equations were largely derived from ambulatory populations with mild to moderate CKD and relatively stable Scr [12]. Additionally, acute changes in the Scr can invalidate conventional estimates of kidney function, where the estimates depend on the assumption that function is at steady state [18]. The Jelliffe equation was developed to estimate GFR in AKI, where kidney function is not in steady state [19]. Bouchard and colleagues demonstrated that the Jelliffe equation, modified by consideration of patient volume status, provided a more reliable and accurate assessment of kidney function when compared with timed urine collections in AKI [20]. While several studies have evaluated CG compared to MDRD estimates in patients with CKD, there is a paucity of data comparing whether alternative GFR estimating equations might alter dosing of drugs in AKI [11,21]. The objective of this study was to compare the theoretical influence of different estimating equations on drug dosing of antimicrobials in patients with AKI.

## 2. Experimental Section

The Program to Improve Care in Acute Renal Disease (PICARD) group included five academic medical centers in the United States. The study was approved by the ethics committees at each participating clinical site. A total of 618 subjects were enrolled over a 31-month period (February 1999 to August 2001), among who 398 required IHD or CRRT. We conducted a retrospective chart review of antimicrobial dosing for a subset of patients from one center in the PICARD data set. Complete descriptions of PICARD methods have been previously published [2,3]. 

AKI was defined differently depending on the baseline Scr. In patients with baseline Scr < 1.5 mg/dL, AKI was defined as an increase in Scr ≥ 0.5 mg/dL, whereas in those with baseline Scr ≥ 1.5 mg/dL and ≤ 5 mg/dL, as an increase in Scr ≥ 1 mg/dL. Patients with a baseline Scr > 5 mg/dL were not considered for study inclusion. Pertinent data elements from PICARD used for these analyses included age, sex, height, weight (to calculate body surface area), daily fluid balance, daily Scr and all dates on which patients received intermittent hemodialysis (IHD) or continuous renal replacement therapy (CRRT).

For inclusion in this study, patients were required to have complete laboratory information and must have received antimicrobials during some dates of enrollment in the PICARD study. We excluded the time period during which patients were on IHD or CRRT, including the days before and after dialysis. Drug dispensation records were retrieved electronically and included antibiotic name, dose, frequency, route, start and stop dates. 

### 2.1. Estimation of GFR Using Cockroft-Gault, MDRD, Jelliffe and Modified Jelliffe Equations

Estimations of CLcr or GFR using CG [17], abbreviated MDRD (age, race, gender and Scr) [10], MDRD adjusted for BSA, Jelliffe [19] and Modified Jelliffe equations [20] were calculated for each patient during each date of admission that they received an antimicrobial agent (Table 1). For the CG equation, total body weight was used if this weight was less than 130% of ideal body weight. If total body weight was greater than 130% of ideal body weight, an adjusted body weight was calculated by adding 40% of the difference between the total and ideal body weights to the ideal body weight. 

Clearances were calculated at the peak and nadir Scr values to describe the severity and resolution of the AKI. The CG equation was used as the reference estimate for the analysis, as the CG equation is the most frequently used equation by pharmacists for drug dosing [18]. Timed urine collections were performed as part of routine medical care for a small subset of patients and the duration ranged from 4 to 24 h.

### 2.2. Evaluation of the Discordance in Drug Dosing Among Estimating Equations

Institutional guidelines from the University of California, San Diego on drug dosing in patients with impaired kidney function were utilized to assess dose appropriateness. These guidelines suggest using the CG equation for drug dosing and are based on modified FDA recommendations. An antimicrobial episode was defined as each day that the patient received the antimicrobial or any time the antimicrobial was altered (e.g., change in antimicrobial dose or frequency). The rate of discordance in drug dosing was calculated as the difference in the number of correctly dosed antimicrobial episodes between the CG and the other estimating equations (Table 1) divided by the total number of antimicrobial episodes (Equation (1)).

(#Correct Episodes CG – #Correct Episodes comparison) × 100 Total #Episodes(1)

We used antimicrobial episodes for calculating discordance since a single patient may receive numerous antimicrobials for varied durations of therapy. Additionally, the potential for error could be different depending on the antimicrobial and dosing range. A chi square test of independence with Bonferroni correction for multiple comparisons was used to assess if there was a difference in number of antimicrobial episodes dosed correctly between estimating equations for each antimicrobial (R 2.8.1). Two-tailed *p*-values < 0.05 were considered statistically significant. 

## 3. Results

A total of 719 antimicrobial episodes from 32 unique patients were included in the analysis. The median age was 49.5 (range 31 to 89) years, 12.5% had CKD and the most common etiology of AKI was acute tubular necrosis (ATN). Demographic characteristics are summarized in Table 2.

**Figure 1:**
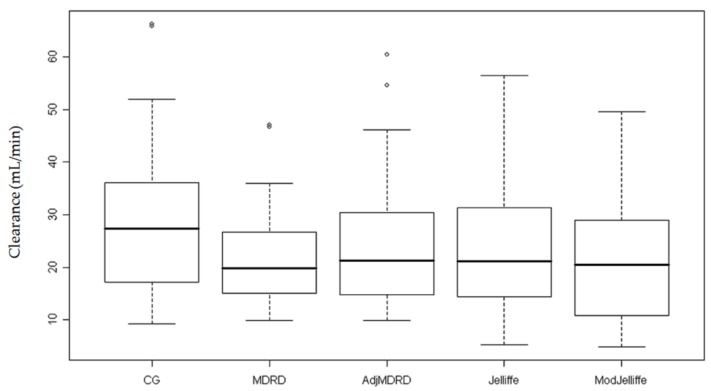
Clearance estimates at peak of kidney injury. This figure depicts the calculated median clearance using the peak serum creatinine value in each estimating equation. The circles represent outlier data points.

**Figure 2:**
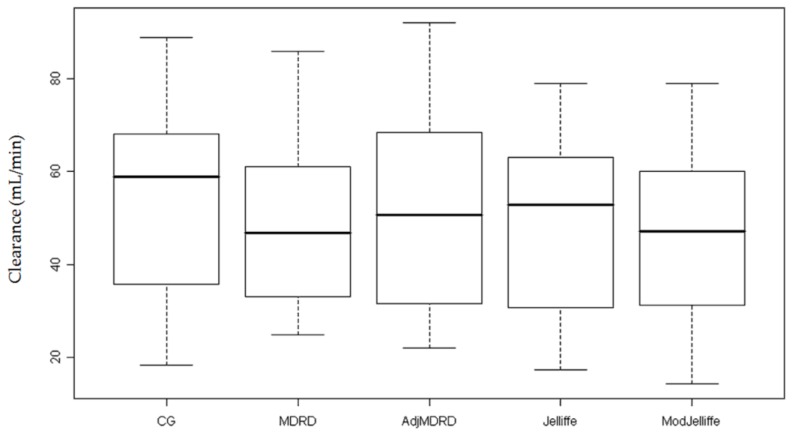
Clearance estimates at recovery of injury. This figure depicts the calculated median clearance at the time of injury recovery using the lowest serum creatinine value in each estimating equation.

Daily Scr values were used to estimate clearance for the entire cohort (Table 3). In order to show the spectrum of AKI, we calculated the median clearance at peak and nadir Scr and this ranged from 19.8 to 27.4 and 46.9 to 58.8 mL/min, respectively (Figure 1 and Figure 2). During the course of AKI, there was a clinically meaningful change in kidney function of approximately 30 mL/min, which would indicate the need for re-evaluation of drug dosing.

### 3.1. Overall Impact on Drug Dosing Based on Individual Equations

Patients received at least one or more of the following antimicrobials whose disposition is influenced by kidney function: ampicillin, cefazolin, ceftazidime, ciprofloxacin, fluconazole, ganciclovir, metronidazole, piperacillin/tazobactam. Of the 719 dosing episodes, the appropriate dose of antimicrobials was administered at most 81% of the time (Table 4). Seventeen patients received a total of 139 episodes of inappropriate doses according to the CG equation, but after removing these inappropriate doses, 30 patients and 580 episodes remained. The discordance rate between the CG equation and the other estimation equations ranged from a minimum of 9% to a maximum of 16% (Table 4).

### 3.2. Breakdown of Impact on Drug Dosing Based on Drug Administered

The most commonly prescribed drugs in our study population were ceftazidime, ciprofloxacin and fluconazole with 69%, 66% and 47% of patients receiving these medications. In the majority of cases, the discordance between estimating equations was statistically significant. The discordance rate for all episodes varied among the antimicrobial agents from 6 to 22% (Table 5). 

The discordance in drug dosing between the CG and the Modified Jelliffe was highest for cefazolin (22%), ganciclovir (20%) and ceftazidime (16%). In patients who were not dosed correctly according to CG, we did not find any episodes of under-dosing. We analyzed the direction of error in dosing in the subset of episodes where the dose was correct based on the CG estimate of clearance (Table 6).

Depending on the frequency of antimicrobial administration, the percentage of over-dosing episodes was as high as 30%. Furthermore, in reviewing the doses administered, excess doses were clinically relevant for some antimicrobials (Table 6).

## 4. Discussion

In our study, we found that patients received an inappropriate dose of antimicrobials in approximately one in six dosing episodes. Almost half of the patients included in this study experienced a dosing error. The change in estimated clearance was clinically significant for the majority of patients, warranting a dosage adjustment of medications. The overall discordance rate between the CG equation and the other estimating equations was between 9% and 16%. Importantly, the difference in clearance between the estimating equations was approximately 8 mL/min or a 30% relative difference. This difference also crossed a cutoff value for our institutional guidelines for dosage adjustment (30 mL/min) resulting in antimicrobial dosing discordance. This presents a clinical challenge since physicians and pharmacists are faced with different kidney function estimates, which ultimately lead to variable doses of critical medications. We found that in cases of discordances between the CG and Modified Jelliffe equations, the dose of antimicrobial administered was supra-therapeutic when using the Modified Jelliffe estimate as the reference point. 

Several factors can be attributed to the challenge of drug dosing in AKI. These include the delayed rise of Scr in response to injury, the accuracy of the various estimating equations in AKI, the lack of therapeutic drug monitoring for several antimicrobials, as well as the lack of published pharmacokinetic information on antimicrobial dosing in patients with AKI not receiving dialysis or hemofiltration. 

Estimating kidney function in AKI remains controversial. Hoste and colleagues have demonstrated that in critically ill patients with normal Scr, urinary excretion of creatinine was markedly reduced [22]. They concluded that using Scr to predict kidney function was insensitive in the critically ill population and advocated for the use measured CLcr [22]. Clinicians often measure CLcr with urinary collections since patients may have an indwelling catheter and the collection can be completed by a nurse. However, urinary CLcrs have been shown to be inaccurate in the critically ill population. Robert and colleagues published results comparing the performance of 30 min urinary CLcr, 24 h urinary CLcr, and CG estimates to inulin clearance in 20 critically ill patients with stable Scr whose mean was 1.8 ± 1.5 mg/dL [16]. The 30 min collection performed similarly to the 24 h collection, but in a subset of patients, urinary CLcr over-predicted GFR by 30–300% [16]. Bragadottir and colleagues demonstrated that urinary CLcr had a low reproducibility compared to measured GFR [23]. Given the limitations of urinary collections in the critically ill population, estimating equations are attractive for routine bedside approximations of GFR. To date there are various studies comparing the ability of estimating equations to accurately predict measured CLcr or GFR in patients with stable renal function [8,9,10,11,12,13,14,15,16,17,24]. Analysis of these studies indicates that the most accurate equation varies according to the population studied [9,15]. Steady state equations such as CG will systematically over-estimate clearance and lead to over-dosing episodes in patients with AKI. Kirwan and colleagues compared the accuracy of the various steady state estimating equations to measured CLcr (4 h collection) in critically ill patients with AKI. They found that the accuracy of the various equations within 50% of measured CLcr to be 68, 78 and 81% for the CG, MDRD and CKD-EPI equations respectively [25]. The performance of these equations was not as good as in the setting of CKD. Poggio and colleagues examined the accuracy of the CG and MDRD equations in estimating GFR compared to measured GFR in hospitalized patients with kidney dysfunction [13]. They demonstrated that the MDRD and CG equations over-estimated GFR and the accuracy of the estimates within 50% of the measured GFR was 49% and 40%, respectively [13]. Bragadottir found that the MDRD, CKD-EPI and CG equations performed poorly when compared to measured GFR in critically ill patients with early AKI with biases of 7.39–11.58 mL/min [23]. This bias is consistent with other studies noting over-estimation of measured CLcr by approximately 6–17 mL/min [25,26]. These steady state equations are problematic for the estimation of kidney function in an intensive care setting or in AKI.

Non steady state equations such as Jelliffe will provide estimates of GFR that are closer to the true clearance [19]. Using data from PICARD, Bouchard and colleagues compared the accuracy of estimating GFR using CG, MDRD, Jelliffe and a modified Jelliffe equation to that of a 24-h measured urinary CLcr [20]. The authors found that among critically ill patients with AKI, traditional estimating equations (CG, MDRD) significantly overestimate kidney function compared to a modified Jelliffe equation adjusted for fluid balance [20]. 

One limitation of this study was the small sample size of 32 patients, as we could retrieve antimicrobial dosing data only on a small subset of patients from the PICARD database. In addition, the retrospective nature of the study is a limitation in capturing the dynamic nature of prescribing and pharmacist consulting on antimicrobial doses. This safety concern was unanticipated but provides strong rationale for electronic algorithms for drug dosage adjustments according to kidney function. Our retrospective study is limited in assessing the validity of the estimating equations for patients with AKI. We utilized the CG estimate as the criterion standard since this is the most commonly used equation for adjusting the doses of drugs [18]. We found discordance in kidney function estimates but we are limited in concluding which equation is most accurate and whether the use of the Modified Jelliffe equation would have resulted in appropriate antimicrobial concentrations. Most clinicians feel that the therapeutic index is wide for many antimicrobials such as penicillins and cephalosporins. However, inappropriate dosing may contribute to the development of super-infections and increased costs. 

Our study did not include antimicrobials in which therapeutic drug monitoring is available. If serum concentration monitoring is available, this guides dosage adjustments and little emphasis is placed on the renal estimating equation. The GFR estimating equations are used to calculate initial doses and subsequent dosing is based on serum concentrations. 

Our study did not include patients on IHD or CRRT. In the setting of dialysis, a fixed clearance is prescribed. However, estimating CLcr from the prescribed effluent volumes may not be accurate since the clearance delivered is frequently less than that prescribed [27]. Dosing guidelines for many drugs in AKI are generally derived from experience in patients with CKD. This may not account for changes in drug metabolism, tubular function or drug transport in the setting of AKI [28,29,30,31]. Applying CLcr estimates from prescribed effluent volume, utilizing dosing guidelines derived from CKD and a lack of available therapeutic drug monitoring may create a potential for under-dosing antimicrobials in a critically ill population receiving IHD or CRRT [32]. 

The KDIGO position statement on drug dosing considerations indicates there is a lack of compelling evidence for the superiority of any one estimating equation for drug dosing [18]. The Acute Disease Quality Initiative (ADQI) recommends the use of short timed urine collections or the modified Jelliffe equation for estimating kidney function in persistent AKI [33]. Further research is needed on the quantification of kidney function in persistent AKI in the critically ill population.

## 5. Conclusions

Critically ill patients with AKI are at risk for significantly increased morbidity and mortality. It is essential that drugs be dosed as accurately as possible to minimize potential adverse effects and improve patient outcomes. The observations from our study indicate that there is discordance in drug dosing when using kidney function estimating equations. Prospective studies evaluating the Modified Jelliffe equation and other strategies for drug dosing in the setting of AKI should be undertaken.

## Figures and Tables

**Table 1:** Equations used to estimate renal function.

Name	Equation
Cockcroft Gault	CLcr = ((140 – age) × weight (kg))/(72 × Scr (mg/dL)) Multiply by 0.85 if female
MDRD	GFR = 186 × (SCr (mg/dL))^–1.154^ × (age (years))^–0.203^ × (0.742 if patient is female) × (1.21 if patient is black)
MDRD adjusted for BSA	GFR = MDRD × BSA / 1.73 m^2^
Jelliffe	(((Volume of distribution × (Scr on day 1 – Scr on day 2)) + creatinine production) × 100/1440/average Scr
Modified Jelliffe	Substitute Adjusted SCr into Jelliffe equation Adjusted SCr = SCr (measured) × Correction Factor Correction Factor = ((admit weight (kg) × 0.6) + Sum (Daily fluid balance))/admit weight × 0.6

CLcr = creatinine clearance, MDRD = modification of diet in renal disease, GFR = glomerular filtration rate, BSA = body surface area.

**Table 2:** Demographics.

Variable	*n* (%) or Median (Range)
Age (years)	49.5 (31–89)
Gender Male Female	14 (44%) 18 (56%)
Weight (kg)	73.9 (45–99)
Height (cm)	169 (152–191)
BSA (m^2^)	1.81 (1.38–2.26)
APACHE III Score *	90 (38–151)
History of CKD	4 (12.5)
Etiology of AKI ATN Nephrotoxicity Multifactorial Hepatorenal Prerenal	14 (44%) 2 (6%) 11 (34%) 3 (10%) 2 (6%)

* APACHE III scores available for 30 patients.

**Table 3:** Clearance estimates.

Parameter	Timed Urine Collection	CG (mL/min)	MDRD (mL/min/1.73 m^2^)	MDRD – Adj BSA (mL/min)	Jelliffe (mL/min)	Modified Jelliffe (mL/min)
CL peak Scr Median (range)	-	27.4 (9.3–66.3)	19.8 (9.8–47.0)	21.2 (9.9–60.4)	21.2 (5.2–56.4)	20.5 (4.9–49.6)
CL Nadir Sc rMedian (range)	-	58.8 (18.4–88.9)	46.9 (24.8–85.8)	50.8 (22.0–92.1)	52.8 (17.3–79.0)	47.2 (14.3–79.0)
Median CL (range)	22.8 (13.4–26.2)	34.4 (9.3–88.9)	28.6 (9.8–85.8)	29.3 (9.9–92.1)	30.3 (4.5–78.9)	26.7 (4.6–78.9)

**Table 4:** Dose appropriateness for all drugs.

Estimating Equation	Number Dosed Correct (%) *n* = 719 episodes (32 patients)	Discordance Rate (%)	Number Dosed Correct (%) *n* = 580 episodes (30 patients)	Discordance Rate (%)
CG	580 (81)	-	580 (100)	-
MDRD	529 (74)	7	515 (89)	11
MDRD BSA	531 (74)	7	526 (91)	9
Jelliffe	531 (74)	7	530 (91)	9
Mod Jelliffe	488 (68)	12	485 (84)	16

**Table 5:** Dose appropriateness for specific antimicrobials.

Drug	# Patients Received (%)	# Correct CG (%)	# Correct Mod-Jelliffe (%)	Discordance Rate (%)	*p* Value
All drugs	-	580/719	488/719	13	<0.001
Ceftazidime	22 (69)	140/200 (70)	107/200 (54)	16	0.009
Ciprofloxacin	21 (66)	164/170 (96)	153/170 (90)	6	-
Fluconazole	15 (47)	104/129 (81)	91/129 (71)	10	-
Metronidazole	11 (34)	52/52 (100)	45/52 (87)	14	-
Cefazolin	7 (22)	31/36 (86)	23/36 (64)	22	-
Ganciclovir	7 (22)	59/92 (64)	41/92 (45)	20	-
Ampicillin	4 (13)	10/16 (63)	9/16 (56)	6	-
Piperacillin/Tazobactam	4 (13)	16/16 (100)	15/16 (94)	6	-

**Table 6:** Correct doses for Cockcroft Gault but overdosing for modified Jelliffe.

Antimicrobial	Number of Patients	Number of Dosing Episodes	Number of Overdosing Episodes	Median Daily Dose (Range)	Median Overdoseper Day (Range)
Acyclovir	2	4	0	2400 mg	0 mg
Ampicillin	3	10	1	3500 mg (3000–8000)	5000 mg
Cefazolin	7	31	7	3000 mg (2000–3000)	1000 mg
Ceftazidime	20	140	33	2000 mg (500–3000)	1000 mg (500–3000)
Ciprofloxacin	24	164	11	500 mg (400–1500)	400 mg (200–500)
Fluconazole	17	104	16	100 mg (50–400)	50 mg (50-100)
Ganciclovir	6	59	18	Oral: 3000 mg (1000–3000) IV: 100 mg (75–400)	Oral: 2000 mg (1000–2000) IV: 110 mg (45–200)
Metronidazole	11	52	7	1500 mg (1000–1500)	500 mg
Piperacillin/Tazobactam	4	16	1	11,250 mg (6750–1,3500)	4500 mg
